# The Endothelial Glycocalyx: New Diagnostic and Therapeutic Approaches in Sepsis

**DOI:** 10.1155/2016/3758278

**Published:** 2016-09-06

**Authors:** Lukas Martin, Patrick Koczera, Elisabeth Zechendorf, Tobias Schuerholz

**Affiliations:** Department of Intensive Care and Intermediate Care, University Hospital Aachen, RWTH Aachen University, Pauwelsstr. 30, 52074 Aachen, Germany

## Abstract

Sepsis is defined as a life-threatening organ dysfunction caused by a dysregulated host response to infection. The endothelial glycocalyx is one of the earliest sites involved during sepsis. This fragile layer is a complex network of cell-bound proteoglycans, glycosaminoglycan side chains, and sialoproteins lining the luminal side of endothelial cells with a thickness of about 1 to 3 *μ*m. Sepsis-associated alterations of its structure affect endothelial permeability and result in the liberation of endogenous damage-associated molecular patterns (DAMPs). Once liberated in the circulatory system, DAMPs trigger the devastating consequences of the proinflammatory cascades in sepsis and septic shock. In this way, the injury to the glycocalyx with the consecutive release of DAMPs contributes to a number of specific clinical effects of sepsis, including acute kidney injury, respiratory failure, and septic cardiomyopathy. Moreover, the extent of glycocalyx degradation serves as a marker of endothelial dysfunction and sepsis severity. In this review, we highlight the crucial role of the glycocalyx in sepsis as a diagnostic tool and discuss the potential of members of the endothelial glycocalyx serving as hopeful therapeutic targets in sepsis-associated multiple organ failures.

## 1. Introduction

Defined as a “life-threatening organ dysfunction caused by a dysregulated host response to infection”, sepsis represents a severe disorder with a devastating mortality exceeding for septic shock in hospitals of about 40% [1]. Although recognized as a disease of modern-day hospitals and critical care medicine, already Hippocrates of Kos mentioned the term sepsis (*ήψις*), which was further illuminated by his succeeding fellows, that is, Semmelweis and Pasteur [2, 3]. Since the new millennium, the definition for sepsis has changed several times; concurrently, the research community demonstrates rapidly new insight into this complex disease [4–6]. The alternating definitions also demonstrate the difficulty in a comprehensive understanding of the complex pathophysiology. The current sepsis definition focuses on organ dysfunction, which is associated with the high mortality [1]. The origin of this organ dysfunction is based on the dysregulated interaction of host response to an infection [1]. Generally, host defence begins with the recognition of pathogens via a set of receptors recognizing pathogen-associated molecular patterns (PAMPs). However, during the last decades, also endogenous ligands have been described as causative agents of tissue injury and cell damage [7]. In contrast to the pathogen-associated molecular patterns (PAMPs), the origin of damage-associated molecular patterns (DAMPs) lies within the host, as tissue damage after major surgery or burns causes liberations of degradation products of the endothelial glycocalyx, such as heparan sulfates [7–10]. Acting as highly potent DAMPs, these glycocalyx fragments trigger the devastating consequences of the proinflammatory cascades in sepsis and septic shock [10]. The present review focuses on the endothelial compartment during sepsis, highlights the important role of the glycocalyx as a diagnostic tool, and discusses the potential of members of the endothelial glycocalyx serving as hopeful therapeutic targets in sepsis-associated multiple organ failures.

## 2. The Endothelial Glycocalyx

### 2.1. Structure

Endothelial cells line the luminal side of blood vessels, thereby modulating the microvascular environment. Towards the tissue, endothelial cells connect to the basement membrane. Endoluminal, the endothelial glycocalyx, coats the endothelial cells and interacts with the blood, thereby regulating microcirculatory flow [12]. This fragile endothelial surface layer has a thickness from 1 to 3 *µ*m and consists of proteoglycans, glycoproteins, glycosaminoglycans (GAGs), and associated plasma proteins, including albumin. GAGs consist of a core membrane-bound protein of the syndecan or glypican families with attached heparan or chondroitin sulfate side chains [13]. Moreover, hyaluronan, a nonsulfated, uncharged GAG, is attached to cell-surface proteins (CD44) and stabilises the glycocalyx structure by exhibiting water-retaining characteristics [14].

### 2.2. Physiological Role of the Endothelial Glycocalyx

The endothelial glycocalyx specifically exhibits crucial roles in the mediation of shear-stress and the associated production of nitric oxide as well as the housing of vascular protective enzymes (e.g., superoxide dismutase) and a wide range of anticoagulant factors (e.g., antithrombin, protein C, and tissue factor pathway inhibitor) [15]. Moreover, the endothelial glycocalyx modulates the inflammatory response by mediating the leukocyte adhesion as well as binding of several inflammatory mediators, such as chemokines, cytokines, and growth factors [12]. Beside these modulating assignments, the endothelial glycocalyx is crucial for maintenance of the vascular barrier [16]. In 1896, Starling described the chain of endothelial cells as an impermeable membrane for proteins. According to this principle, the endothelial cells separate the interstitium, a plasma layer low in proteins, from the protein-rich intravascular space. However, this principle did not consider the reduction in fluid extravasation by the endothelial glycocalyx. In conclusion, with the current knowledge about the role of the endothelial glycocalyx, the Starling principle has to be revised, considering the following aspects: venous reabsorption, the amount of capillary filtration, and the opposition to capillary filtration [16].

### 2.3. Alteration of the Endothelial Glycocalyx during Sepsis

**Figure 1:**
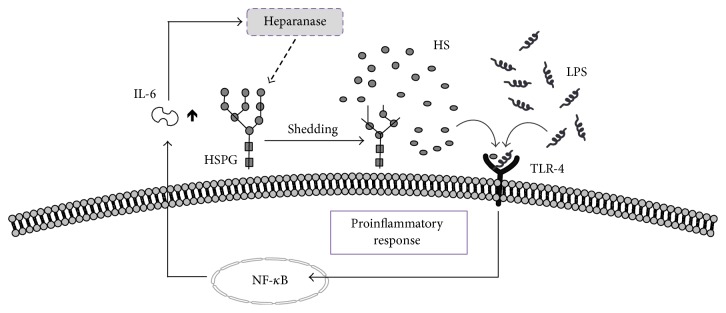
Model of proinflammatory response induced by heparanase. Heparanase cleaves and solubilizes heparan sulfate (HS) fragments from their proteoglycan (HSPG) within highly sulfated regions. Analogue to LPS, HS fragments then signal through MyD88-dependent receptors, of which TLR-4 is one, and this leads to NF-kappaB cleavage and activation. NF-kappaB-dependent upregulation leads to the release of cytokine production including interleukin-6 (IL-6). Cytokines are involved in activating heparanase, thereby enhancing the devastating circle of an inflammatory response.

Alteration in the composition of the glycocalyx after exposure to an inflammatory insult is one of the earliest features during sepsis. Destruction of the glycocalyx leads to capillary leakage, accelerated inflammation, platelet aggregation, coagulation, and loss of vascular tonus [17]. By expressions of adhesion molecules like intercellular adhesion molecule 1 (ICAM1) or vascular cell adhesion molecule 1 (VCAM1), endothelial cells enable leukocyte adherence, rolling, and migration [18]. Notably, neutrophil granulocytes are recognized as Janus-faced actors during sepsis. They have a fundamental role in the clearance of pathogens, but neutrophil activation is also associated with tissue damage. In this setting, secretion and activation of sheddases harm tissue integrity by degradation of the extracellular matrix and components of neutrophil extracellular traps (NET) act as DAMPs [19, 20]. Heparanase represents one of these enzymes and is activated by proinflammatory cytokines, for example, reactive oxygen species [21]. Today, only one human form is known, the heparanase-1 [22]. This highly specific enzyme is an endo-*β*-glucuronidase, which sheds heparan sulfate side chains from their proteoglycan within highly sulfated regions [22] (Figure 1). Thereby, after cleavage of the 65-kDa heparanase to its active 50-kDa, heparanase liberates circulating heparan sulfates, which act as highly potent DAMPs [10, 23]. Recently, we showed that circulating heparan sulfate in the serum of septic shock patients induces a strong proinflammatory response in cardiomyocytes, hence causing cardiac mitochondrial dysfunction [9, 10].

Besides enabling leukocyte migration, endothelial activation enhances the inducible nitric oxide synthase, which causes peripheral blood pooling by vasodilatation. Additionally, blood coagulation is shifted towards a procoagulatory state, and vascular permeability is increased due to tight-junction loosening, causing extravasation of tissue and plasma proteins into the surrounding tissue [24, 25]. These alterations seem locally reasonable, as they allow for bottling pathogens up for immunological clearance. Systemically, this reaction has serious implications on circulation and hence on tissue nourishment and oxygenation. In conclusion, the injury of the endothelial glycocalyx causes the clinical appearance of critically ill septic patients, who present generalized oedema and concurrent intravascular hypovolemia, low blood pressure, and high pulse frequency.

## 3. Markers of Glycocalyx Degradation as Diagnostic Tools

As a result of its unique position directly between the blood and the vessel wall, the endothelial glycocalyx plays a pivotal role in microvascular physiology, in particular by regulating vascular endothelial permeability, vascular tone, and coagulation [26]. During inflammation caused by sepsis or major trauma, the glycocalyx becomes “activated,” which appears to be directly involved in a widespread of endothelial damage, hence contributing to microvascular dysfunction [12]. Thus, there is a strong pathophysiological rationale for targeting markers of endothelial damage during sepsis. Up to now, over 1.200 original articles as well as several reviews have been published evaluating markers of endothelial activation in critically ill patients [27]. The following paragraph aims to discuss the potential of the newly investigated but promising markers of endothelial damage, such as syndecan-1, heparan sulfates, heparanase, endocan, and angiopoietins as diagnostic tools in sepsis.

### 3.1. Syndecan-1

Circulating levels of syndecan-1 are related to endothelial damage and glycocalyx degradation. Rehm and colleagues investigated syndecan-1 levels in arterial blood of patients undergoing surgery of the ascending aorta. During early reperfusion after global ischemia with circulatory arrest, they reported a transient 42-fold increase in syndecan-1 [28]. Furthermore, electron microscopy in guinea pigs showed a shedding of the glycocalyx with a consecutive loss of syndecan-1 after ischemia and reperfusion (I/R) [28]. In fact, in addition to these findings, syndecan-1 correlates with coagulopathy and increased mortality in sepsis patients [29]. In this study, levels of syndecan-1 have been evaluated in 104 patients suffering from severe sepsis or septic shock, in 28 patients after major abdominal surgery and in 18 healthy young volunteers without any signs of infection. Levels of syndecan-1 were markedly elevated in the sepsis and the surgery group, compared with the control group. Notably, septic patients showed significantly higher levels than patients belonging to the surgery group [29]. Moreover, there was a strong correlation between levels of IL-6 and syndecan-1 in both sepsis and surgery group [29]. In addition, another study with 20 patients shows a significant increase in circulating syndecan-1 on sepsis onset [30]. However, in the plasma of nine healthy male volunteers undergoing endotoxemia (0.5 ng/kg/hour infusion of* E. coli* LPS), syndecan-1 plasma levels did not increase after 4 and 6 hours. The authors conclude that endothelial disruption and damage observed in patients with severe sepsis cannot be fully reproduced in human experiments, since unsafe and ethically unacceptable doses of LPS would therefore be needed [30]. However, the applied endotoxemia did influence the endothelium as evidenced by an early decline in protein C and a late increase in tPA [30]. Likewise, another prospective observational study with 20 patients suffering from septic shock and 20 healthy adults volunteers likewise showed a significant increase in syndecan-1 content in plasma of septic patients, compared to controls [31].

### 3.2. Heparanase

**Figure 2:**
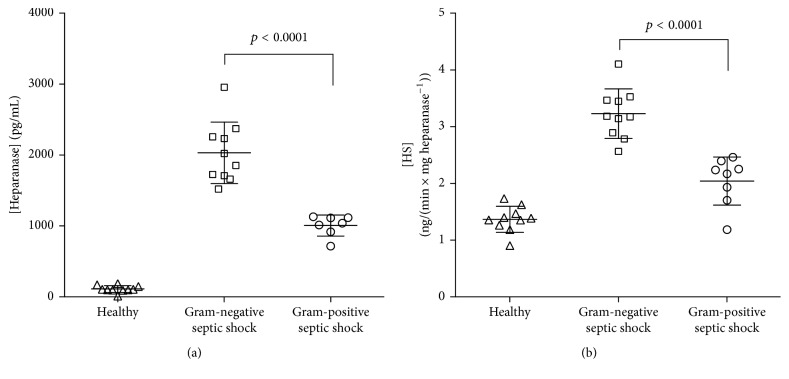
Heparanase level (a) and activity (b) in human sepsis (*n* = 18) and healthy volunteers (*n* = 10). Patients with Gram-negative (*n* = 10) septic shock show higher levels of heparanase and higher heparanase activity, compared to those suffering from Gram-positive (*n* = 8) septic shock (modified from [11]).

**Figure 3:**
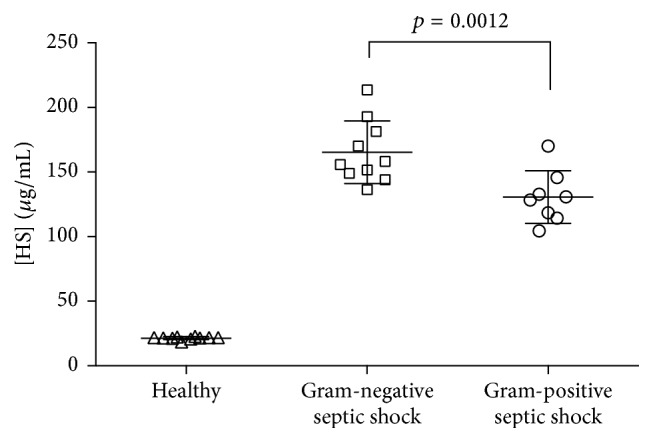
Heparan sulfate levels in human sepsis (*n* = 18) and healthy volunteers (*n* = 10). Patients with Gram-negative (*n* = 10) septic shock show higher levels of heparan sulfates, compared to those suffering from Gram-positive (*n* = 8) septic shock (modified from [10]).

The elevated expression of heparanase has been reported in several studies evaluating human malignancies. Heparanase expression correlates with enhanced local and distant metastatic spread, increased vascular density, and reduced postoperative survival [32, 33]. Moreover, heparanase levels are elevated in the urine and plasma of patients with diabetes and correlate with blood glucose levels [34]. Two studies indicate that heparanase expression is elevated during sepsis-associated pulmonary [35] and renal [36] failure. However, these measurements are limited to tissue levels in certain organs [35]. Therefore, we recently measured heparanase level and activity in the plasma from 18 patients suffering from Gram-negative (*n* = 10) or Gram-positive (*n* = 8) septic shock as well as in healthy humans (*n* = 10). We found a significantly higher level and activity of plasma heparanase in septic shock patients compared to healthy volunteers (Figure 2). Of note, there was a significant difference of heparanase levels between the strains of infection, with a significant higher heparanase level and activity in patients with Gram-negative septic shock [11]. As shown in Figure 3, these findings accompanied with significant higher levels of circulating heparan sulfates in patients with Gram-negative septic shock [10].

### 3.3. Heparan Sulfate

Several studies identified elevated levels of circulating heparan sulfate fragments in critically ill patients [9, 10, 29, 31, 37, 38]. Nelson and colleagues measured heparan sulfate levels in plasma obtained from patients admitted to the intensive care unit with septic shock as well as from matched control patients scheduled for neurosurgery. Median levels of heparan sulfates were fourfold increased in septic shock and were threefold higher in nonsurvivors (90 days study period). Thereby, levels of heparan sulfate correlated with levels of interleukin-6 and interleukin-10. Similarly, the already mentioned study with 104 patients suffering from severe sepsis or septic shock, 28 patients after major abdominal surgery, and 18 healthy controls shows higher levels of heparan sulfate in the sepsis group and the surgery group, compared to the control group [29]. Surprisingly, in comparison to the syndecan-1 levels (see above), the heparan sulfate levels were higher in the surgery group, compared to the sepsis group [29]. Recently, we identified a difference in heparan sulfate levels according to the type of bacterial infection (Figure 3) [10]. We sampled serum from 18 patients suffering from Gram-negative (*n* = 10) or Gram-positive (*n* = 8) septic shock as well as from healthy humans (*n* = 10). As expected, heparan sulfate levels were significantly higher in patients with septic shock compared to healthy volunteers (Figure 3). Notably, there was a significant difference of heparan sulfate levels between the strains of infection, with significantly higher heparan sulfate levels in patients with Gram-negative septic shock [10].

### 3.4. Endocan

Endocan is a soluble endothelial proteoglycan, known to be released during inflammatory response [39]. As such, endocan is considered as a promising biomarker of endothelial dysfunction in sepsis [12]. In 150 patients suffering from sepsis or septic shock, endocan plasma levels showed a highly predictive value to diagnose patients with sepsis and septic shock and revealed prognostic information for 30-day and 6-month all-cause mortality [40]. Using venous occlusion plethysmography, Cox and colleagues showed that endocan is related to endothelial dysfunction in humans in vivo [41]. They investigated the endothelial function in 17 healthy male volunteers before and 4 h after the administration of 2 ng/kg LPS. Plasma levels of endocan significantly increased after LPS administration. Furthermore, there was a significant correlation between the increase in plasma endocan levels and the attenuation of vasodilatatory responses to acetylcholine [41]. Similarly, another study with 78 patients showed that endocan plasma levels at day 0 are in patients with bacteremia compared to those without bacteremia, but neither CRP levels nor PCT levels at day 0 are different between the two groups [42]. Moreover, endocan levels <2.54 ng/mL at admission seem to be highly predictive of a respiratory failure presence at day 3 after admission [43]. Using another threshold of 6.2 ng/mL in 63 patients admitted to the intensive care unit with sepsis, the sensitivity and specificity of endocan for predicting mortality were 75% and 84%, respectively. Measurement of endocan at intensive care unit admission revealed higher levels in nonsurvivors than in patients still alive 10 days later [44]. The results of the studies mentioned above suggest that, in septic patients, endocan blood levels are related to the severity of illness and the outcome of the patient and may represent a useful marker of endothelial dysfunction in sepsis and septic shock.

### 3.5. Angiopoietins

Angiopoietins (Angs) belong to a novel class of angiogenetic growth factors, playing several roles during inflammatory response [45]. Ang-1 is crucial for the stability of blood vessels, whereas Ang-2 destabilizes vascular integrity and increases vascular permeability [45]. In this way, Ang-2 reflects the breakdown of the vascular barrier in critically ill patients [27]. Up to now, more than 10 studies investigating Ang-2 as a novel biomarker in sepsis and septic shock have been published [27]. Overall, these studies show that Ang-2 is increased in septic shock [46]. Notably, Kümpers and colleagues report an independent association of circulating Ang-2 levels with 30-day survival after adjustment for APACHE II score, SOFA score, and serum lactate levels [47]. Moreover, Ricciuto and colleagues observed that serial measurements of Ang-2 are associated with 28-day mortality and multiple organ dysfunction (MOD) score [48]. In this study, sepsis survivors had lower daily levels of Ang-2 than nonsurvivors [48]. However, up to now, a cut point or threshold of circulating Ang-2 allowing differentiation of patients with infection or sterile inflammation or stratification of patients with respect to sepsis severity based on baseline or serial serum Ang-2 concentrations remains still uninvestigated [27].

## 4. Therapeutic Strategies

As discussed above, the endothelial glycocalyx is extensively involved in sepsis-related inflammatory response and organ dysfunction. Thus, strategies aiming at protecting or repairing glycocalyx damage reveal promising therapeutic targets in sepsis therapy [12, 15]. In this context, especially hydrocortisone, albumin, and adequate fluid resuscitation have been investigated during the last decades. These studies have shown that the therapeutic potential of these drugs may be, at least partly, based on the protection of the endothelial glycocalyx damage [17, 49]. Thus, the following subsection aims to outline established and experimental therapies to protect or repair glycocalyx damage during sepsis.

### 4.1. Hydrocortisone

Hydrocortisone is known to exhibit strong anti-inflammatory effects in several pathophysiological settings including I/R injury [16]. Thereby, glucocorticoids attenuate glycocalyx degradation by suppressing cytokine and chemokine release as well as reducing the migration of inflammatory cells and mast cell degranulation [16]. Furthermore, hydrocortisone exhibits protective effects against I/R injury by mediating nontranscriptional activation of eNOS [50]. Since the first step of endothelial injury after ischemia consists in a disruption of the glycocalyx [51], Chappell and colleagues investigated the role of hydrocortisone in shedding of the endothelial surface layer after I/R in an isolated heart model [52]. The administration of hydrocortisone reduced shedding of syndecan-1, heparan sulfate, and hyaluronan and consecutively attenuated postischemic oxidative stress and transudate formation. Moreover, electron microscopy revealed a mostly intact glycocalyx after hydrocortisone treatment [52]. A prospective study with 91 patients undergoing cardiac surgery showed that perioperative stress doses of hydrocortisone attenuate systemic inflammation and improve early outcome [53]. However, these findings seem to be limited to a predefined risk group of cardiac surgery patients, since a recent randomized controlled trial with 4494 patients reports no benefit of the use of dexamethasone, regarding the 30-day incidence of major adverse events, compared with placebo [54]. Similarly, the role of hydrocortisone in sepsis therapy remains controversial. Although clinical evidence exists that glucocorticoids improve vasopressor efficacy, it is uncertain whether patients benefit regarding the outcome [55]. Except a postulated protective effect of corticosteroids on glomerular glycocalyx [56], up to now, no data exist on the impact of steroids on the glycocalyx during sepsis.

### 4.2. Fluid Resuscitation

Fluid resuscitation is one of the fundamental principles for the management of sepsis [1]. However, there is emerging evidence that the type and dose of fluid crucially affect the outcome [57]. Several clinical studies have shown that hypervolemia has detrimental influences on patient outcome, including cardiopulmonary complications, anastomotic insufficiency, and mortality [56, 58]. A pilot study with elective surgery patients shows that hypervolemia increases the release of atrial natriuretic peptide (ANP) and causes enhanced shedding of the endothelial glycocalyx [49]. ANP is known to induce rapid shifts of intravascular fluid into the interstitium. Thereby, elevations of ANP preceded those of cytokines and coincided with or even preceded shedding of the glycocalyx in patients undergoing heart surgery [59]. Indeed, the integrity of the glycocalyx and its interaction with plasma-derived proteins, in particular albumin, is mainly influenced by the perioperative fluid management [16]. Ex vivo investigations showed that albumin prevents fluid extravasation in the heart more effectively than crystalloid or artificial colloid. Notably, this effect is independent of colloid osmotic pressure, rather based on an interaction of albumin with the endothelial glycocalyx [60]. In this way, even very low concentrations of albumin maintain endothelial barrier function [61]. Despite the supposed beneficial effect of albumin in experimental studies, in patients with severe sepsis, albumin replacement in addition to crystalloids did not improve outcome [62]. These negative results may be partly explained by the fact that a colloid only behaves, as first predicted by Starling, if the glycocalyx is undamaged and there is a volume deficit [63]. In fact, if the endothelial glycocalyx is damaged, oncotic pressure gradients play a minimal role because a large amount of protein-rich plasma translocate into the interstitial space, thereby minimizing the oncotic pressure gradient [64].

### 4.3. Heparanase Inhibition

Heparanase, a heparan sulfate-specific glucuronidase, mediates the onset of renal dysfunction and lung injury during sepsis [35, 36]. The structure of unfractionated heparin (UFH) is comparable to heparan sulfate but has higher N- and O-sulfate contents [65]. UFH is known as potent heparanase inhibitor [66]; however, the potent anticoagulative activity limits the therapeutic use as anti-inflammatory drug [65]. Schmidt and colleagues studied in a model of sepsis-induced renal and pulmonary injury the potential of nonanticoagulant N-desulfated re-N-acetylated heparin (NAH) as a competitive heparanase inhibitor [35, 36]. Heparanase inhibition by NAH prevented endotoxemia-associated glycocalyx loss and neutrophil adhesion and, accordingly, attenuated sepsis-induced acute lung and renal injury and improves survival in mice subjected to polymicrobial sepsis [35, 36]. Notably, heparanase inhibition seems to be protective also after sepsis onset. Delayed heparanase inhibition 24 h after the onset of sepsis attenuated pulmonary endothelial hyperpermeability, suggesting that heparin is a lung-protective intervention even in established sepsis [35]. Heparin and its derivatives can bind histones through electrostatic interaction, which show pivotal inflammatory mediators in sepsis-associated acute lung injury [67]. In an aspiration model of ALI, induced by intratracheal instillation of hydrochloric acid (HCl), NAH improved the lethality rate, blood gas, MPO activity, lung oedema, and pathological score. However, UFH tended to aggravate the injury due to haemorrhagic complications [67]. These reported data suggest that heparanase inhibition, especially with NAH, may be a promising therapeutic approach in sepsis therapy. However, more experimental and clinical studies are needed to verify this strategy.

### 4.4. Synthetic Antimicrobial Peptides

The growing relevance of antimicrobial peptides is because of their promising capacity to act as additive drugs in times of increasing antibiotic resistance [68]. Thereby, antimicrobial peptides decrease inflammatory response, kill bacteria, and stimulate innate immunity [69]. Synthetic antimicrobial peptides have been designed based on the limulus-anti-LPS-factor to bind the lipid A-moiety of LPS [70]. However, in addition to a protection against Gram-negative bacteria, they attenuate inflammation and improve survival in Gram-positive bacterial, viral, and mixed infections in vitro and in experimental settings in vivo [10, 71, 72]. Of note, cellular attachment of enveloped viruses was shown to be decreased by a strong interaction between these synthetic antimicrobial peptides and glycocalyx-bound heparan sulfates [71]. Thus, peptide binding to and neutralization of circulating highly potent heparan sulfates may be the underlying mechanism for controlling inflammation [10]. In this way, synthetic antimicrobial peptides offer the unique opportunity to cope with both, PAMPs and DAMPs, and exhibit their activity in both infectious and sterile inflammation, by this dual characteristic [10]. The underlying mechanism seems to be a charge-dependent alteration in the secondary structure of both, PAMPs and DAMPs [11, 71, 73]. Moreover, we recently reported that the treatment of septic mice with the synthetic antimicrobial peptide 19–2.5 lowers levels of plasma heparanase and circulating heparan sulfate and reduces heparanase activity, compared to untreated control animals [11]. Additionally, mRNA levels of heparanase in several organs are downregulated in septic mice treated with peptide 19–2.5, compared to untreated control animals [11]. We also tested the ex vivo addition of peptide 19–2.5 to plasma of septic shock patients. As already mentioned above, plasma heparanase level and activity are elevated in septic shock and the addition of peptide 19–2.5 decreases heparanase activity [11]. These findings have been underlined by isothermal titration calorimetry, which revealed a strong exothermic reaction between peptide 19–2.5 and heparanase, indicating a direct Coulomb interaction between the positive charges of the peptide and the negative groups or heparanase [11]. Thus, synthetic antimicrobial peptides seem to be a potential anti-inflammatory agent in sepsis by interaction with members of the endothelial glycocalyx.
